# Nanostructured Fibers Containing Natural or Synthetic Bioactive Compounds in Wound Dressing Applications

**DOI:** 10.3390/ma13102407

**Published:** 2020-05-23

**Authors:** Alexa-Maria Croitoru, Denisa Ficai, Anton Ficai, Natalia Mihailescu, Ecaterina Andronescu, Claudiu Florin Turculet

**Affiliations:** 1 Faculty of Applied Chemistry and Materials Science, University Politehnica of Bucharest, Gh. Polizu St. 1-7, 011061 Bucharest, Romania; croitoru.alexa@yahoo.com (A.-M.C.); denisaficai@yahoo.ro (D.F.); anton.ficai@upb.ro (A.F.); ecaterina.andronescu@upb.ro (E.A.); 2 Academy of Romanian Scientists, Spl. Independentei 54, 050094 Bucharest, Romania; 3 Laser Department, National Institute for Laser, Plasma & Radiation Physics, Atomistilor St. 409, 077125 Magurele, Romania; 4 Faculty of Medicine, Carol Davila University of Medicine and Pharmacy, Eroii Sanitari St. 8, 050474 Bucharest, Romania; claudiu.turculet@umfcd.ro; 5 Emergency Hospital Floreasca Bucharest, Calea Floreasca St. 8, 014461 Bucharest, Romania

**Keywords:** electrospinning, electrospun nanofibers, wound dressing, natural/synthetic substances

## Abstract

The interest in wound healing characteristics of bioactive constituents and therapeutic agents, especially natural compounds, is increasing because of their therapeutic properties, cost-effectiveness, and few adverse effects. Lately, nanocarriers as a drug delivery system have been actively investigated and applied in medical and therapeutic applications. In recent decades, researchers have investigated the incorporation of natural or synthetic substances into novel bioactive electrospun nanofibrous architectures produced by the electrospinning method for skin substitutes. Therefore, the development of nanotechnology in the area of dressings that could provide higher performance and a synergistic effect for wound healing is needed. Natural compounds with antimicrobial, antibacterial, and anti-inflammatory activity in combination with nanostructured fibers represent a future approach due to the increased wound healing process and regeneration of the lost tissue. This paper presents different approaches in producing electrospun nanofibers, highlighting the electrospinning process used in fabricating innovative wound dressings that are able to release natural and/or synthetic substances in a controlled way, thus enhancing the healing process.

## 1. Introduction

Skin wounds are a type of injury which disrupt the structure of the tissue and lead to an abnormal behavior. Wound healing of both acute and chronic skin injuries is a very complicated process, and today’s treatments face great challenges in clinical practice. One of the essential medical methods of treating chronic wounds is the use of suitable topical dressings with extraordinary properties in order to increase the healing process of wounds [1,2,3].

Latterly, researchers have focused on developing modern nanofiber-based dressings that can mimic the native dermal extracellular matrix (ECM) and have the role of establishing and maintaining an optimal environment for wound repair [4]. These nanofibers have the ability to release active substances, such as natural or synthetic antimicrobials or different agents with intrinsic bacteriostatic and/or bactericidal activity able to stimulate the healing process. For an effective design of a functional wound dressing, certain features must be fulfilled: wound type, wound healing time, physical, mechanical, and chemical properties of the scaffold are important factors [1,5,6,7]. An ideal wound dressing should provide bacterial protection that allows fluid exchanges, a moist environment (removing excess exudate), and optimal healing environment, be non-toxic, non-allergenic, and cost effective [8,9], biocompatible without any rejection and/or inflammation, and help accelerate re-epithelialization. The porous architecture should be interconnected for the transportation of oxygen and nutrients and a good cell adhesion should be facilitated by having a large surface area [10,11]. Also, a good strength of the dressing is required due to various mechanical deformations and dynamic loadings [12].

In order to obtain such suitable dressing materials, both the biomaterial composition and the processing technology play important roles. Thus, biomaterials such as natural polymers (collagen, chitosan, keratin, etc.) or synthetic polymers (poly(lactic acid), polyethylene oxide, polycaprolactone, poly(lactic-co-glycolic acid), etc.) could be suitable dressing materials for skin regeneration [12,13].

In recent years, the incorporation of various therapeutic substances (natural or synthetic) into such structures could accelerate the healing process of the wounds and regeneration of the lost tissue providing synergistic effect for wound healing applications [14,15]. The use of natural compounds in wound healing processes has been widely demonstrated in the literature due to their therapeutic properties, cost-effectiveness and low adverse effects [3,16]. Nonetheless, these products have low bioavailability and are not stable in the long term, leading to a slow healing process [17,18,19]. Recently, the design and fabrication of novel bioactive nanofibrous mats via electrospinning (the most effective, facile and cost-efficient method generating a variety of polymeric 2D or 3D scaffolds for artificial body tissue implantation or other applications) has attracted more attention [20,21,22]. The novel electrospun nanofibrous mats should act like an effective physical barrier that protects the open wound from further contamination with exogenous microorganisms [23,24].

This review paper provides a brief overview of the advantages of electrospun nanofibrous membranes applied as wound dressings. In addition, the incorporation of natural and/or synthetic substances into electrospun nanofibers aiming to prevent skin infections as well as enhance the healing process is highlighted. The technology limitations, research challenges, and future trends are also discussed.

## 2. Different Methods of Producing Nanofibers

Over the last decade, a great interest has been given to the production of nanofibrous materials, due to their various and useful applications in biomedical and industrial fields. In order to fulfil this demand, researchers have developed various techniques, as shown in Table 1, that are used to produce polymers into different types of nanostructured dressings in order to perform a rapid healing of the wound and to provide a good efficient drug loading [24,25,26,27,28].

The drawing process is similar to dry spinning in fiber industry in which very long fibers are formed and is applicable for viscoelastic materials that can undergo strong deformations while being cohesive enough to support the stresses developed during pulling. Nanofibers are formed one by one. Thus, this method is discontinuous, with small productivity, and limited to laboratory scale [26]. Self-assembly is a technique where small components organize in a concentric manner through non-covalent forces: (hydrophobic forces, hydrogen bonding and electrostatic reactions) in order to form nanofibers. It requires standard laboratory equipment and only uses specific polymers. Although similar to phase separation, it is time consuming in terms of the processing of continuous polymer nanofibers. It is easy to obtain smaller nanofibers, but it is a complex and extremely elaborate process with reduced productivity and no control of fiber dimension [25]. The phase separation process is very simple and consists of polymer dissolution, gelation, extraction using a different solvent, freezing, and freeze drying under vacuum. The process is limited to the laboratory scale and needs a minimum equipment. So far, only a few polymers were used to form nanofibers. The process is time consuming and the obtained nanofibers are small [28]. In template synthesis, polymers, metals, semiconductors, or ceramics are formed using nonporous membrane with numerous tubular pores (5–50 mm thickness). The technique cannot make one-by-one continuous nanofibers, and only a few micrometers of length are obtained. The pore size of the membrane can determine the diameter of these fibers [24,29].

Although, these techniques are used to obtain nanofibers with high surface area in an easy way, the resulting structures do not meet required conditions for an ideal wound dressing material. They do not have the required mechanical stability, making the control of the porosity difficult, the resulting shapes are not very uniform, and the loading efficiency low when using these techniques [30].

## 3. The Electrospinning Technique

Among all these techniques, electrospinning is considered the most used process for fabrication of ideal electrospun nanofibers for wound dressings, since the technique is suitable for delivering various biological agents long-term to local tissues at the wound site [31,32]. Electrospinning is an economical, simple, and facile technique with a specific approach using electrostatic forces to generate uniform nanofibers/nanobots from polymer solutions. Based on the research studies reported in the literature [25,33], by using the same experimental set-up, the diameter of fabricated fibers may vary between ∼3 nm and 6 µm, reaching up to several meters in length [34,35].

**Figure 1:**
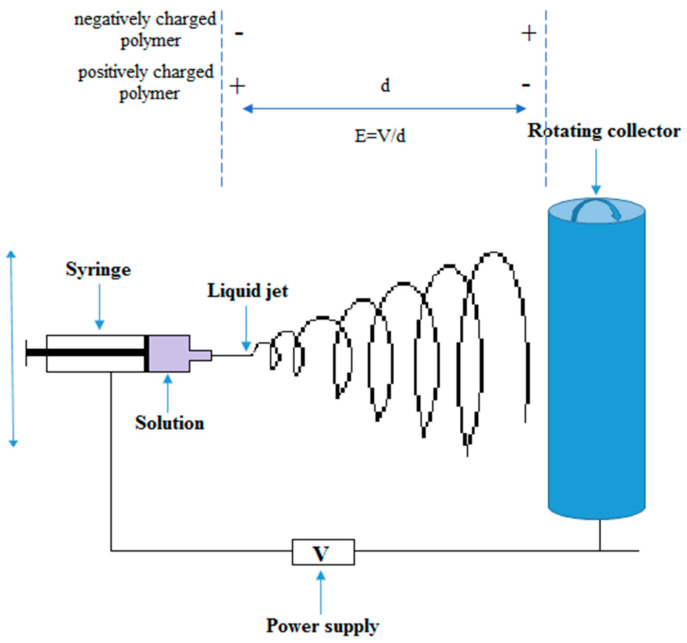
General view of electrospinning process.

The electrospinning process is illustrated in Figure 1. A polymer is dissolved into a solvent (or is melted) before the solution is placed into a glass capillary or a syringe. An electrostatic force is applied between the drum (or the collector, plane or cylinder) and the syringe needle. When the voltage (electrostatic field) is applied, flowing polymer (in molted or dissolved form) is extruded from the needle and the droplet/fibers exposed to electric field begins to form fibers [20,36,37]. Depending on the overall charge of the polymer molecule (chemical nature), the electric field is applied. If the polymer is negatively charged, the needle should be negative while the collector should be positive. In this way, the polymer solution is electrically forced to deposit on the collector. Certainly, if the polymer is positively charged, the electric field will be opposite (the needle will be positive and the collector negative). Equation (1) is used to calculate the applied electric field in electrospinning technique [38].

(1) $$E=\frac{V}{d}$$

where *E* is the applied electric field in kV/m, *V* is the voltage supplied to the needle and collector in kV, and *d* is distance between needles and collector in m.

In order to fabricate these electrospun nanofibers/mats, numerous synthetic and natural polymers, and polymers loaded with functional materials, such as nanoparticles, hydrophilic and/or hydrophobic active agents, enzymes, growth factors, etc., have been successfully electrospun into nanofibers for wound healing purposes.

The loading of biomolecules and bioactive agents into electrospun fibers, the high surface area and 3D porous structure, provide the possibility to reduce the minimum required drug dosage which implies less side effects [36,39,40]. The physical and mechanical properties along with well-controlled degradation rates and biocompatibility of the base polymer, make biodegradable polymeric materials ideal candidates for developing scaffolds for tissue engineering [41].

**Figure 2:**
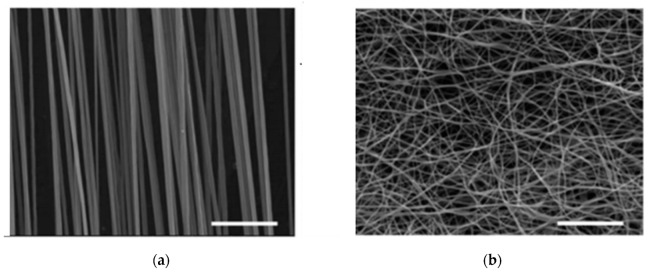
Different types of fibers: (**a**) aligned fibers and (**b**) randomly oriented fibers (adapted from [43]) (Copyright permission: Direct cryopreservation of adherent cells on an elastic nanofiber sheet featuring a low glass-transition temperature O. Batnyam, S. Suye and S. Fujita, RSC Adv., 2017, 7, RSC Advances DOI: 10.1039/C7RA10604A).

Different techniques of electrospinning are used in order to fabricate fibers with a broad range of properties that can be used in various biomedical and industrial applications. Coaxial-electrospinning is a technique for fabricating core-shell electrospun nanofibers using two concentrically aligned (co-axial) nozzles (the drug is placed in the inner jet and the polymer is in the outer tubing), thus obtaining a prolonged delivery of the drug to the wound site [42]. Fiber orientation (Figure 2) [43], morphology, and uniformity represent another important matter that needs to be controlled through modification of the parameters of the electrospinning process (molecular weight, viscosity, surface tension, conductivity, and concentration of polymer solution-polymer solution parameters; electrical voltage delivered, temperature, the flow rate of solution, needle-collector distance, and rotational speed of the drum-setup parameters; humidity, pressure, and type of atmosphere-ambient conditions) [24,44,45,46].

Electrospun nanofibers have been considered as applicable materials for wound dressing because of their high surface-area-to-volume ratio and swelling properties [27] and due to the similarity of the structure with ECM, which encourage cell adhesion and proliferation [3,47]. Furthermore, electrospun nanofibers can be loaded with a high amount of bioactive medical substances (up to 40% loading) [48], with antimicrobial and antioxidant activity that can enhance the wound healing. The porous structure of these matrices could facilitate the adsorption of wound exudates, gaseous and nutrients exchange, and prevent bacterial infection [49,50]. Compared to traditional nanofiber drug-delivery systems, electrospun nanofiber dressings could allow for shorter response times and more precise control over the release rate of therapeutic agents for wound healing. Moreover, electrospun nanofibers have a better coverage and protection against bacterial infections and expansive surface area that enables efficient loading of drugs. Therefore, such advantages make these advanced biologically active nanofibers highly attractive in the biomaterials field [51,52,53].

## 4. Use of Synthetic Antibiotics/Substances in Wound Healing

The long-lasting antimicrobial properties of materials containing different synthetic substances such as antibiotics were extensively studied and their efficacy in treating life-threatening burn-related injuries or/and infections was demonstrated [54,55,56]. Abdallah [57] investigated the antibacterial activity of amoxicillin incorporated into PEG electrospun nanofibrous scaffolds against common microorganisms which can cause skin and wound infections. The morphological and FTIR analysis confirmed an ideal concentration of 35% w/v of PEG solution in chloroform and no chemical changes in PEG and amoxicillin after electrospinning. *In vitro* studies of PEG loaded amoxicillin showed inhibition against *S. aureus*, *S. pyogenes,* and *E. coli*, but no inhibitory effect on *P. aeruginosa* due to its resistance to amoxicillin. As a conclusion, the PEG/amoxicillin nanofibrous scaffold can easily release and diffuse amoxicillin in the agar medium, which can be similar to wet wound dressing.

**Figure 3:**
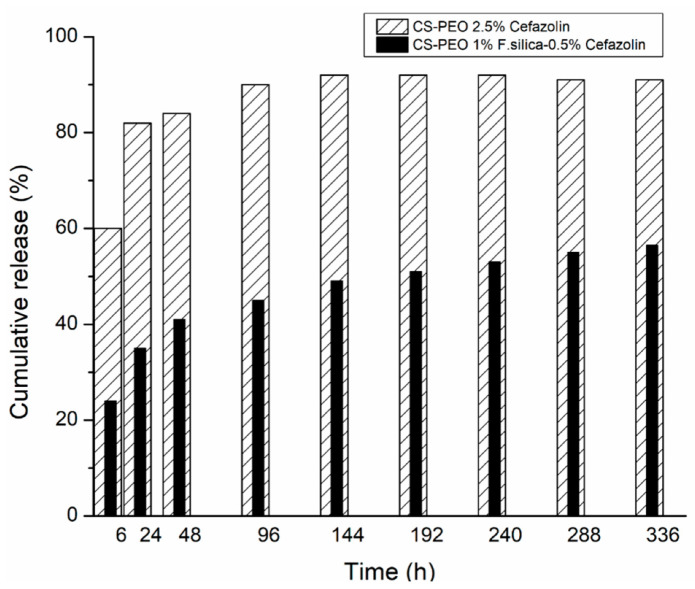
The release diagram for CS-PEO 2.5% Cefazolin and CS-PEO 1% F. silica-0.5% Cefazolin nanofibrous mats (adapted from [58]).

The antimicrobial effect against both *S. aureus* (SA) and *E. coli* (EC) was demonstrated using chitosan-polyethylene oxide (CS-PEO) nanofibrous materials loaded with 2.5% cefazolin (CF), 1% fumed silica (F. silica)–0.5% cefazolin (F. silica-CF). The release behavior of CF from CS-PEO materials loaded with 2.5% CF and 1% F. silica-0.5% CF was monitored for 15 days (Figure 3). The results showed an increased release of CF within first 24 h for CS-PEO mats loaded with 2.5% CF and a burst release in the first 6 h for CS-PEO mats loaded with 1% F. silica-0.5% CF nanoparticles, but afterwards the drug release was gradually made. Because the amount of CF released from the fibers, containing 2.5% drug, was much higher than the fibers loaded with 0.5% drug, the latter being more suitable for biomedical applications because the release of the drug was made more slowly. The nanocomposite membrane was evaluated in vivo on wounded skin rats, as treatment group and the results showed that the wounds of the rats were almost entirely healed after 10 days. The wounded skin of the control group was only wrapped by comfeel glue and after 10 days the skin of the rats still had some bleeding. These results demonstrated the performance of CS-PEO-F. silica-cefazolin nanofibrous mat in wound repair [58].

Li [54] used the electrospinning method to develop novel thermoresponsive wound dressing materials that can be removed easily without secondary injuries. The composite fibers were composed of poly(di(ethylene glycol) methyl ether methacrylate) (PDEGMA) and poly(L-lactic acid-co-ε-caprolactone) (P(LLACL) (different ratio) and loaded with ciprofloxacin (Cip). The cyto-compatibility tests of the composite fibers showed adhesion and proliferation of L929 fibroblasts cells. By reducing or increasing the temperature, cells could easily be attached to and detached from the fibers. The release profile and antibacterial activity were also evaluated in vitro, and the results showed a sustained release of Cip over more than 160 h. Moreover, the drug-loaded fibers inhibited the growth of *E. coli* and *S. aureus. In vivo* tests were performed on rats and the results showed that PDEGMA/P(LLACL)/Cip fibers had increased wound healing properties compared with commercial gauze or P(LLACL)/Cip fibers. After 21 days, the wound was largely scab-free and mostly filled with regenerated skin and hairs. These results show that the composite fibers loaded with Cip can act as potent wound dressing materials. 

Recently, Yang [59] studied the release profile, antibacterial activity and cytotoxicity of chitosan (CS), PVA and graphene oxide (GO) loaded with Cip and CipHCl for wound dressing applications. At 4 h, CS/PVA/GO/Cip and CS/PVA/GO/CipHCl nanofibrous membrane showed a release ratio of 18.7% and 30% respectively. After 12 h, the release profile reached a steady stage and over 168 h, the release ratio for the two nanofibrous membranes was 96.5% (CS/PVA/GO/Cip) and 62.1% (CS/PVA/GO/CipHCl), respectively. The two membranes also showed enhanced antibacterial activity against *E. coli*, *S. aureus*, and *B. subtilis* and excellent cytotoxicity with melanoma cells after the addition of the drug. These findings suggest that the obtained nanofibrous membranes can be further developed for production of wound dressings.

The release profile, cytotoxicity and antibacterial activity of PVA/CS and PVA/CS/Tetracycline hydrochloride (TCH) electrospun mats were evaluated for wound dressing. The release behavior was realized in PBS solution and the results showed an initial burst release of TCH in the first 2 h, but afterwards the drug release was gradually made. Also, PVA/CS/TCH nanofibrous mats showed an effective antibacterial activity against *E. coli*, *S. epidermidis*, and *S. aureus* and a good cytocompatibility, indicating that PVA/CS/TCH electrospun mat can be tough as an antibacterial wound dressing [60].

Li [61] fabricated a multifunctional scaffold material loaded with mupirocin and lidocaine as a novel wound dressing and studied the release profile and antibacterial activity against *S. aureus*, *E. coli*, and *P. aeruginosa*. The first layer of the scaffold contained polycaprolactone (PCL) and the second was formed from CS. The results showed a burst release of lidocaine of 66% within an hour and only 5% release for mupirocin. Moreover, the nanofibers exhibited high antibacterial activity and was nontoxic to fibroblasts.

In a recent study, Ghalei and collaborators [62] prepared a core-sheath wound dressing material from PVA and zein nanoparticles (NPs) loaded with diclofenac (DLF) using single jet electrospinning method. The optimum loading efficiency of DLF in zein NPs was of 47.80%. The release profile of PVA/zein NPs/DLF and zein NPs/DLF nanofibers were studied and in the first 8 h, and approximately 36% of DLF was released from the NPs. After three days, 80% of the encapsulated drug was released, reaching a plateau. In contrast, the release profile of DLF from PVA/zein NPs was only 20% in the first 8 h, followed by a sustained release in the next five days, reaching nearly 80%. These results demonstrated that adding zein NPs into the PVA nanofibers ensure a slower and controlled release of the drug in order to prolong release time and reduce the burst release of the drug. The biocompatibility test and cytotoxicity of PVA, PVA/zein NPs, and PVA/zein NPs/DLF nanocomposite dressings were also examined and the obtained data revealed that all samples are biocompatible and non-toxic to fibroblast L929 cells. The addition of NPs to the structure of PVA nanofibers led to better cellular proliferation. These findings make PVA/zein NPs/DLF nanofiber a promising drug-delivery system with great potential for wound healing applications.

PCL (single) and PCL/gelatin (binary) electrospun mats loaded with 5% Ketoprofen (Ktp) were fabricated using electrospinning technique. *In vitro* release studies showed that the single PCL/Ktp mat exhibited a burst release of the drug of ~90% that reached a plateau after approximately 12 min. In contrast, the binary PCL/Ktp mat exhibited a continuous and sustained drug release for about four days, demonstrating that the latter is much more efficient in releasing the drug in a controlled manner. Cytotoxicity studies were performed with L929 fibroblast cells. The electrospun mats containing Ktp exhibited no toxic effect on the cells and showed enhanced attachment and proliferation of L929 fibroblast cells. The developed electrospun mats demonstrated potential wound dressings [63].

Poly(L-lactic acid) (PLA) nanofibers containing 10, 20, and 30 wt % ibuprofen were successfully prepared and the release profile and cytotoxicity were evaluated. Controlled release of ibuprofen was dependent on temperature and initial concentration of the drug. At 37 °C the release of ibuprofen from PLA was higher compared to room temperature. The higher ibuprofen release was realized by 30 wt % ibuprofen loaded PLA scaffolds at 37 °C (0.25 mg at 336 h). *In vitro* analysis revealed that 20 wt % ibuprofen loaded PLA nanofibrous scaffolds significantly increased viability and proliferation of human epidermal keratinocytes (HEK) and human dermal fibroblasts (HDF). PLA/ibuprofen scaffolds were also evaluated *in vivo* in nude mice to determine the ability of these scaffolds to promote skin regeneration. The obtained data showed that 20 wt % ibuprofen bandages exhibited significantly greater blood vessel formation and reduced wound concentration confirming the biocompatibility of this scaffolds [64].

In the wound healing process, complex cellular activities involving structural proteins, growth factors etc. play a key role in tissue repair and wound care. The sustained release of macromolecules with antimicrobial and inti-inflammatory properties from electrospun fibers can be achieved for days to months, which is beneficial for wound healing [48,65]. Sebe [66] studied the antimicrobial and healing effect of all peptides optimized (APO) monomer-coated PVA nanofiber patches in the treatment of uninfected and *A. baumannii*-infected wounds on mice. The wound healing of APO patches was compared to patches without antibiotic colistin after three days, and the results revealed an accelerated wound healing, reduced wound size and wound bacterial load in the case of APO patches. The *in vivo* antimicrobial effect was statistically significant when compared to colistin nanofiber effect, by using only one tenth of the active pharmaceutical ingredient. These results suggest that the APO-patches can be further developed as a cost-effective treatment to skin and blast injuries and battlefield burns.

Diabetic patients have lower immunity system therefore they are more prone to infection in wounds. Growth factors in tissue engineering technologies represent a solution to wound healing problems. Novel biomimetic drug delivery systems containing PLGA/Gelatin 70:30 and PLGA/Gelatin 50:50 nanofibers loaded with gentamicin sulfate (GS) were fabricated. The scaffold was further immobilized with recombinant human epidermal growth factor (rhEGF). The antibacterial activity and wound healing efficiency of the nanocomposite carrier was investigated *in vitro* and *in vivo* on diabetic C57BL6 mice with dorsal wounds, while releasing gentamicin and rhEGF in a controlled manner. The presence of rhEGF in the nanofibrous scaffold hasten the wound-healing and the scaffolds exhibited continuous proliferation for 12 days. The antibacterial activity of gentamicin sulfate loaded PLGA/Gelatin 70:30 and PLGA/Gelatin 50:50 nanofibrous scaffolds were explored against *S. aureus* M 0092. Both nanofibrous scaffolds inhibited bacterial growth. Such results support the applicability of the nanocomposite in rapid wound healing eliminating infections [67].

Among many other applications, metal oxide nanoparticles such as TiO_2_, MgO, and ZnO have also been studied for their antimicrobial properties [68,69,70,71,72,73,74]. In particular, zirconium dioxide (ZrO_2_) has significant antibacterial and antifungal activities, but not many papers have been published about its antimicrobial activity. Aydogdu [75] used electrospinning technique to fabricate polyurethane (PU)/ZrO_2_ and PU/zeolite nanofiber mats and examined their antimicrobial properties and in vitro cytotoxicity and biocompatibility. Antimicrobial analysis demonstrated that the tested materials inhibited growth and reduced viability of *C. albicans* and *S. aureus* cells. In contrast, PU/ZrO_2_ and PU/zeolite nanofiber mats demonstrated very low inhibitory effect for *P. aeruginosa* cells. Increasing the concentration of PU, the biocompatibility was raised without affecting the antimicrobial ability of the scaffolds. Also, increasing the ration of zeolite and ZrO_2_, cell proliferation and viability had increased significantly. Thus, these nanofibers provide a novel alternative as an antimicrobial material suitable as a wound dressing.

## 5. Use of Natural Substances in Wound Healing

Because of the well-known side effects of antibiotics and synthetic substances on the body, latterly there has been a great interest in using natural substances instead of synthetic antibiotics. Moreover, researchers are facing a concerning problem in terms of multidrug resistant bacteria that emerge as a result of continuous administration of antibiotics. Therefore, in recent years, great interest has been paid to the production of new antimicrobial alternatives which involve the use of natural substances that can be used for the treatment of skin infections [27,76]. The researchers sought to develop different modern wound dressings materials made of nanostructure fibers that can be loaded with natural substances such as phenolic compounds or plant extracts and investigated their release behavior, antibacterial, antioxidant and anti-inflammatory activities in order to hurry the healing process of the wounds [77,78].

Amima [79] fabricated a promising nanostructure dressing from polyurethane (PU) loaded with 5% β-sitosterol and used it to reduce inflammations and to help in wound healing. β-sitosterol possess anti-inflammatory and antioxidant properties while polyurethane has biomedical uses and increases epithelial growth. The biocompatibility of pure PU scaffold and β-sitosterol/PU nanofibrous scaffold was inspected on mouse fibroblast cells. The resultant nanofibers were non-cytotoxic. Therefore, they can be further developed to be used in biomedical applications, especially as efficient wound dressings.

Chitosan, a natural polysaccharide, produced by partial N-deacetylation of the natural polymer chitin with great biodegradability and antibacterial effect, has been considered a good wound healing material [80,81]. Also, chitosan has the property of accelerating the reepithelization and regeneration of the skin. Thus, in combination with natural antimicrobial substances, the wound healing process is accelerated [82]. Figueira and collaborators studied the release profile, cytotoxicity, antimicrobial and anti-inflammatory activities of a bilayer electrospun membrane (EBM) used as a skin replacement. The top layer of the membrane had the role of barrier against external threats and contained hyaluronic acid and PCL and the bottom layer comprised CS and zein loaded with salicylic acid (SA). The obtained membranes were analyzed, and the results revealed good mechanical properties and controlled water loss. The biodegradability of the EBM demonstrated a weight loss of ~10% of its initial weight after 7 days, these results influencing the release profile of SA from EBM of ~16% for 5 days. The cytotoxicity was investigated on NHDF cells. The EBM exhibited cell migration, adhesion and proliferation, enhancing the wound healing process. Bactericidal activity of EBM was evaluated on *S. aureus* stains revealing inhibitory effect of ~99%. These results show suitable properties for EBA to be used as a wound dressing [83].

Ficai [84] investigated the antimicrobial and antifungal activity of CS-based hydrogels loaded with usnic acid (UA). The antimicrobial activity was tested against *C. albicans*, *S. aureus*, and *P. aeruginosa*. The results indicated that samples containing UA exhibited a better antimicrobial activity against *C. albicans* compared to pure CS. The viability of the strains decreased after 24 h. In the case of *S. aureus* and *P. aeruginosa*, the antimicrobial activity is lower for CS/UA, which indicates that these systems are not very toxic to this bacterial strain. Nevertheless, these results are promising because good anti-microbial activity is safe for the beneficial microorganisms existing in the human body. The nanofibrous materials containing PCL, CS, or CS-caffeic acid conjugate (CSCA) had been investigated in wound healing applications. The antimicrobial activity against *S. aureus*, initial cell attachment and proliferation of the material was analyzed. The results showed a better antimicrobial effect of PCL/CH and PCL/CSCA fibrous mats and higher initial cell attachment and cell proliferation in PCL/CSCA fiber material (83%) compared to the PCL (66%) and PCL/CS fiber material (76%). In addition, PCL/CSCA fibrous mat was non-toxic to the NHDF-neo cells. Based on these results, the PCL/CSCA fibrous mat can be a good candidate for wound dressing applications [85].

Recently, the electrospinning technique was used by Wutticharoenmongkol [86] to fabricate cellulose acetate (CA) nanofiber mats loaded with gallic acid (GA) (10 to 40 wt %) and investigate their potential in wound dressing application. The release profile was carried out in acetate buffer solution (pH 5.5) or normal saline (pH 7.0) at 32 and 37 °C. The maximum release capacity of GA was observed from the fiber mats containing 20 and 40 wt % GA with 97% and 71% release in acetate buffer and 96% and 81% into normal saline solution. Also, GA-loaded CA fiber mats showed antibacterial activity against *S. aureus*, making this material suitable for wound dressing applications.

Gandhimathi [87] studied the role of silk fibroin (SF)/vitamin E (VE)/curcumin (Cur) loaded into poly(L-lactic acid)-co-poly-(ε-caprolactone) (PLA/PCL) nanofibers to increase cell proliferation and collagen secretion for application of skin tissue engineering. SF is a natural protein biopolymer with wound healing effect that enhances collagen biosynthesis and VE is an antioxidant that acts as a skin barrier with antitumor activity. Also, Cur is a natural polyphenolic substance with great antioxidant, antitumor, antibacterial, anti-inflammatory and wound-healing effects. The release profiles of 1%, 5%, 10%, and 20% w/w Cur from the nanofibrous scaffolds revealed an initial burst release during the first 15 h, followed by gradual and sustained release of drugs. The maximum released percentage of Cur-loaded nanofibrous scaffolds were about 53%, 68%, 76%, and 85%, respectively. PLA/PCL/SF/VE/Cur promoted cell adhesion and proliferation of human dermal fibroblast cells. The addition of SF into nanofibrous scaffold can lead to an improved secretion of collagen matrix. The findings of this paper indicate that the electrospun biocomposite nanofibrous scaffolds are biocompatible and nontoxic to the surrounding tissue and accelerate wound healing in skin tissue regeneration.

Chrysin (5,7-dihydroxyflavone) (Chr) is a natural flavonoid found in many plant extracts with a wide variety of biological properties such as anti-cancer, anti-inflammatory and antioxidant activities. Deldar [88] studied the antioxidant, anti-inflammatory, cytotoxicity effect and release profile of Chr (5% and 15%) loaded PCL/PEG nanofibrous mats for wound healing applications. PCL/PEG containing 5% w/w Chr revealed a higher release profile (~25 µg) than PCL/PEG containing 15% w/w Chr (~43 µg), after three days. The fibers also exhibited cytoprotective effect towards human HFF-1 fibroblast cells (80% viability), antioxidant and anti-inflammatory properties. Thus, the obtained nanofiber mats could represent a future innovative wound care product.

The anti-inflammatory and antibacterial activity was demonstrated *in vitro* by using electrospun nanofibers from a natural silk fibroin protein (SFP) combined with polyvinylpyrrolidone (PVP) blended with baicalein (BAI), (a flavone isolated from the roots of *Scutellaria baicalensis* and *Scutellaria lateriflora*) against *S. aureus*. The obtained nonwoven mats inhibited the growth of bacteria strains with the maximum amount of release of approximately 64.8% within 24 h of contact with water-based environment. The same nanomaterial was evaluated *in vivo* on mice with a created wound area inoculated with 1 × 107 CFU/mL of *S. aureus*. The results demonstrated a significant enhance in the wound healing process using SFP/PVP/BAI fiber mat (four days of reduction as compared to the untreated group) [89].

*In vitro* and *in vivo* studies were performed for evaluation of antimicrobial and antifungal properties and drug release behavior of electrospun PCL/polystyrene (PS) nanofibrous samples containing chamomile. The drug release of chamomile from optimized PCL/PS nanofiber mat took place gradually after 48 h. The *in vivo* studies showed that the samples loaded with 15% chamomile extract were efficient in wound healing after 14 days post-treatment periods, revealing the formation of epithelial tissue [90].

Bromelain is a mixture of proteolytic enzymes derived from the stem of pineapple and usually used in burn treatments. The release profile and cytotoxicity of CS (2% and 4% w/v)/bromelain nanofiber material were investigated. The results showed a better release profile and low cytotoxicity for CS (2% w/v)/bromelain. Also, the burn healing effect of the nanofibrous material was studied in the induced burn wounds in rats for 21 days and the results indicated a better efficiency in healing burn skin for CS (2% w/v)/bromelain nanofiber [17].

Charernsriwilaiwat [91] investigated the release profile, cytotoxicity, a ntioxidant and antibacterial properties of CS-ethylenediaminotetraacetic acid/polyvinyl alcohol (CS-EDTA/PVA) nanofiber mats loaded with *Garcinia mangostana* (GM) extracts (1, 2, and 3 wt % α-mangostin). The release profile indicated a rapid release of α-mangostin within 60 min (~80%) followed by a sustained release in the next 7 h (~90%). The cytotoxicity of CS-EDTA/PVA/GM extracts was investigated on normal human foreskin fibroblast (NHF) for 24 h demonstrated that the nanofiber mats were non-toxic at concentrations of 1–5 mg/ml. Fiber mats loaded with GM extracts exhibited antibacterial activity against *S. aureus* and *E. coli*. The nanofibrous matrix was tested also *in vivo* on rats and the results demonstrated a complete recovery of the wounds within 11 days for all treatments, with the fastest healing produced by the 3 wt % α-mangostin fiber mats dressing.

It is known that antioxidant, antimicrobial and anti-inflammatory activity of propolis comes from the presence of flavonoids, flavones, phenolic acids and their derivatives. Propolis is considered to be the most potent natural antibiotic with an important biological activity. The characterization of aqueous and ethanolic extracts demonstrated also stronger antibacterial and antiviral activities [36]. The antibacterial, antiviral and cytotoxicity activity of three layers fiber materials containing propolis extract and cross-linked carboxymethyl starch (CL-CMS) were discussed by Sutjarittangtham [92]. The top and the base layers included (PVA) nanofibrous material and the middle layer contained poly(vinyl pyrrolidone) (PVP)/propolis or PVP/propolis/CL-CMS. The amount of propolis differs from 0% w/v, 1% w/v, 3% w/v and 5% w/v in one group and the amount of CL-CMS varied from 0–15% w/v in another group. The antibacterial activity was tested against *S. aureus*, *S. epidermidis*, *B. cereus*, methicillin-resistant *S. aureus* (MRSA), and *P. aeruginosa*. The results of the analyzes showed that the highest antibacterial activity is at 5% w/v of the propolis from the fiber mats. Regarding cytotoxicity and antiviral activity, it was found that when increased the propolis concentration the CD50 was decreased.

Lin [93] studied the effect of grape seed extract (GSE)-loaded SF/polyethylene oxide (PEO) nanofibrous mats on skin fibroblasts and induced oxidative stress. The grape seed extract from nanofibers can be released in a controlled way without modifying the morphology of SF/PEO nanofibers. 3% GSE-loaded mats promoted cell proliferation and were demonstrated to be safe and non-toxic. The incorporation of GSE into SF/PEO significantly increased the antioxidant capacity of the nanofibers. These results indicated the performance of GSE-loaded SF/PEO composite nanofibers in skin repair, tissue regeneration and wound healing.

The antioxidant and antimicrobial activity of a wound dressing material consisting of polysaccharide nanofiber mats loaded with naturally olive leaf extract using electrospinning technique was evaluated. Due to the resistance of numerous bacteria to antibiotics, researchers tested the obtained material against different bacteria strains. These antimicrobial profile demonstrated that the developed electrospun mats inhibited the growth of *S. aureus*, *E. coli*, *Enterococcus faecalis*, and *P. aeruginosa*. Also, the release rate of phenolic compounds was measured and the results indicated a burst release of 75% of herbal extract in the first hour, followed by an extended release of the remaining 15% in the next 24 h [94].

An asymmetric electrospun membrane was obtained using electrospinning technology. The top layer was manufactured with PCL and the bottom layer was manufactured with CS and loaded with Aloe Vera (AV) and had the role of improving the bactericidal activity of the membrane and enhancing the wound healing process. The cytotoxic profile of the membranes was investigated over one, three, and seven days and the obtained data demonstrated that all membranes did not induce any cytotoxic effect on NHDFs cells and moreover, fibroblast cells were able to proliferate on the surface of nanofibrous network. The antibacterial activity was evaluated, and the results showed that the top layer of the membranes acts as protective barrier against *S. aureus* and *E. coli.* These findings reveal the performance of these asymmetric membranes in wound repair [95].

Many essential oils such as thyme, lavender, lemongrass, etc. are considered suitable matrix materials for wound dressing applications due to their antifungal, antioxidant and anti-inflammatory properties [96]. Miguel [97] investigated the release profile, cytotoxicity, and antibacterial properties of a bilayer electrospun asymmetric membrane (EAM) loaded with Thymol (THY). Thereby, the top layer contained silk fibroin (SF) and poly(caprolactone) (PCL) and had the property of a physical barrier at the wound site and the bottom layer was produced with SF and hyaluronic acid (HA) and loaded with THY with the property of enhancing the wound healing process. The release process of THY from the nanofibers, at pH 5 and 8, showed a burst release in the first 8 h after immersion in PBS, followed by a gradual release up to 24 h. Such results can be explained by the high surface to volume ratio of the nanofibers that helps the diffusion of THY in the release media. The cytotoxic profile of the membranes was also evaluated over one, three, and seven days. The results revealed that the membrane could promote cell adhesion, proliferation, and spreading. The antibacterial properties were evaluated against *S. aureus* and *P. aeruginosa* and the results revealed that the top layer acts as a protective barrier for both bacterial models. All these results indicate future perspectives in using these nanofibers in wound healing applications.

Mouro [98] fabricated novel electrospun PCL/PVA/CS mats loaded with 5% w/w Eugenol (EUG), using W/O and O/W emulsions, for wound dressing applications. The release profile of EUG from electrospun fiber mats was investigated and the results revealed a rapid release of EUG during the first 8 h and afterwards the release enhanced gradually over the next days (up to 120 h). The antibacterial of PCL/PVA/CS/EUG mats demonstrated bacteriostatic effect inhibiting the growth of S. aureus (92.43% and 83.08% inhibition rates) and P. aeruginosa (94.68% and 87.85% inhibition rates). In addition, the cytotoxic profile of PCL/PVA/CS and PCL/PVA/CS/EUG fiber mats from W/O emulsions showed no cytotoxicity on human dermal fibroblasts NHDF for at least seven days. Thus, these findings are considered a promising strategy to develop novel electrospun dressings that can treat wound infections.

Table 2 presents the loading behavior and properties of synthetic and natural active substances into different organic supports. Given the fact that a large number of articles about electrospinning are published every year, the number of publications on fibers produced by electrospinning containing a variety of natural products is still low, but is expected to rise further in the coming years [76].

## 6. Conclusions and Future Perspectives

Skin injuries still remain a challenge for the development of dressing materials. Over the last decade, a great interest has been given to the production of nanofibrous materials, due to their various and useful applications in many fields, including wound healing. Various techniques including drawing, self-assembly, phase separation and template synthesis, have been developed to produce polymers into different types of nanostructured dressings, but the resulting structures do not meet the required characteristics/performances for an ideal wound dressing material. Currently, the production of electrospun fibers have been considered suitable for the development of the new class of multifunctional and bioactive dressings because of their high surface-area-to-volume ratio, porosity, bio-inspired architecture that resembles the natural ECM, which supports cell adhesion and proliferation, and capacity to deliver various biological agents long-term to local tissues at the wound site. The presence of bioactive agents (antibiotics, natural substances, macromolecules, etc.) into electrospun dressings has been explored to prevent the skin infections and to enhance the healing process of the wound. In recent years, a great interest has been placed on using natural compounds in wound healing processes due to their therapeutic properties, cost-effectiveness, and few adverse effects. Moreover, bacteria develop resistance to most synthetic antimicrobial agents, in particular antibiotics, that emerge as a result of their continuous excessive administration.

However, despite the encouraging results obtained during the last years to support the development of electrospun dressings, there are still several challenges until electrospun nanofibers can be translated into practical healthcare applications. Primarily, electrospinning has a low production rate that can be an issue that limits the use of electrospun fibers in clinical aspect. Important key factors must be optimized when different active agents are loaded into nanofibers in order to overcome their burst release and low delivery performance. Because large amounts of solvents are used, “green” solvents or even solvent-free methods need to be developed. It would also be ideal for the dressing material to serve as a temporary ECM and degrade in order to allow the cells to fill the space left behind. For example, CS could be used as suitable material. We concluded that the majority of the preliminary studies conducted in vitro are demonstrating encouraging results, but more attention should be focused on in vivo studies, possibly on humans, where realization of clinical trials are of great importance to evaluate the impact and importance of nanofiber technology in healthcare and to enhance the quality of life on patients. Thereby, future research and experimental trials are required in order to establish better control over the physical and mechanical properties, chemical resistance and thermal stability, along with release behavior of electrospun fibers. The physicochemical properties of the nanofibers can be improved through combination between different techniques of nanofiber production and surface modification methods, such as heat and plasma treatment, or chemical grafting. Moreover, co-axial electrospinning, in-situ nanofiber deposition of solution, blow spinning, etc. are advanced spinning methods used to obtain complex nanofiber configurations. Recently, a great interest has been given to centrifugal spinning technique because of its feasible scalability. By combining electrospinning with 3D printing, skin tissue constructs for promotion of wound healing can be fabricated. The combination of electrospinning with additive manufacturing (AM) is already under examination for regenerative medicine applications. For example, melt electrospinning is becoming more interesting for the scientists due to the ability of fabricating fine fibers without any use of solvents.

In the near future, new developments of complex therapeutic fibers that can exploit the properties of various natural substances is also challenging. *In vitro* and *in vivo* tests demonstrated cell migration, proliferation, and accelerated wound healing of electrospun dressings based on natural substances. With the continuous progress of this technology, new studies can be expected, demonstrating the performance of improved electrospun dressings for wound healing processes. With all these new developing technologies, regulatory and economic issues need to be taken in consideration for the use of responsive and adaptive dressings in clinical trials.

## Figures and Tables

**Table 1:** Comparison of different nanofiber fabrication methods [24,25,26,27,28,29].

Method	Control on Fiber Dimension	Advantages	Disadvantages
Drawing	no	− simple process, − simple equipment.	− difficulty in controlling the fiber diameter, − not scalable, − small productivity.
Self-assembly	depends on the precursors	− easy to obtain smaller nanofibers (with diameter of a few nm and length of a few microns).	− complex process, − difficulty in controlling the fiber diameter, − strongly dependent on the nature of materials.
Phase separation	no	− simple procedure, − improved mechanical properties by increasing polymer concentration, − simultaneous presence of nano and macro architecture.	− limited range of materials, − difficulty in controlling the fiber diameter, − not scalable.
Template synthesis	yes	− wide range of materials, − using different templates, the diameter of fibers can be easily changed.	− it cannot make continuous nanofibers, − it is not possible to create complex morphologies because of the lack of templates.
Electrospinning	yes	− cost effective, − thinner diameters of fibers, − continuous fibers, − wide range of materials, − adjustable porosity of electrospun structures, − variety of shapes and sizes, − diversity of assembly organization from 1D to 2D and even 3D materials.	− jet instability, − beads formation, − low production rate, − capillary clogging, − solvent recovery issues, − scalable.

**Table 2:** Synthetic and natural bioactive substances incorporated into organic supports and their properties.

Support Materials.	Active Agent	Activity	Ref.
**Synthetic Substances**			
poly(D,L-lactide-co-glycolide) acid	amoxicillin	antimicrobial	[57]
chitosan-polyethylene oxide	cefazolin fumed silica-cefazolin	antimicrobial release profile wound healing	[58]
poly(di(ethylene glycol)methyl ether methacrylate poly(L-lactic acid-co-ε-caprolactone)chitosan poly(vinyl alcohol) poly(vinyl acetate) graphene oxide	ciprofloxacin ciprofloxacin HCl	release profile antimicrobial wound healing cytotoxicity	[54,59]
poly(vinyl alcohol) chitosan	tetracycline hydrochloride	antimicrobial release profile cytotoxicity	[60]
polycaprolactone chitosan	mupirocin lidocaine	antimicrobial release profile	[61]
poly(vinyl alcohol) zein nanoparticles	diclofenac	release profile cytotoxicity	[62]
polycaprolactone gelatin	ketoprofen	release profile cytotoxicity	[63]
poly(L-lactic acid)	ibuprofen	release profile cytotoxicity wound healing	[64]
poly(vinyl alcohol)	all peptides optimized colistin	antimicrobial wound healing	[66]
poly(D,L-lactide-co-glycolide) acid	rhEGF Gentamicin sulfate	release profile antimicrobial wound healing	[67]
**Natural Bioactive Substances**			
polyurethane	β-sitosterol	cytotoxicity anti-inflammatory	[79]
hyaluronic acid polycaprolactone chitosan zein	salicylic acid	release profile cytotoxicity antimicrobial anti-inflammatory	[83]
chitosan	usnic acid	antimicrobial antifungal	[84]
poly(ε-caprolactone) chitosan	caffeic acid	Antimicrobial cytotoxicity	[85]
cellulose acetate	gallic acid	release behavior antimicrobial	[86]
poly(L-lactic acid)-co-poly-(ε-caprolactone) silk fibroin	vitamin E curcumin	Cytotoxicity release profile	[87]
poly(ε-caprolactone) polyethylene glycol	chrysin	antioxidant anti-inflammatory cytotoxicity release profile	[88]
silk fibroin protein poly(vinyl pyrrolidone)	baicalein	anti-inflammatory antibacterial release profile wound healing	[89]
poly(ε-caprolactone) polystyrene	chamomile	antibacterial antifungal release profile wound healing	[90]
chitosan	bromelain	release behavior cytotoxicity wound healing	[17]
chitosan ethylene diamino tetra acetic acid polyvinyl alcohol	α-mangostin	release profile cytotoxicity antioxidant antibacterial wound healing	[91]
poly(vinyl alcohol) poly(vinyl pyrrolidone) cross-linked carboxymethyl starch	propolis	antibacterial antiviral cytotoxicity	[92]
polyethylene oxide silk fibroin	grape seed extract	release behavior antioxidant cytotoxicity	[93]
polysaccharide	olive leaf extract	antioxidant antimicrobial release profile	[94]
polycaprolactone chitosan	aloe vera	cytotoxicity antimicrobial	[95]
Silk fibroin polycaprolactone hyaluronic acid	thymol	release profilecytotoxicity antimicrobial	[97]
polycaprolactone poly(vinyl alcohol) Chitosan	eugenol	release profile antimicrobial cytotoxicity	[98]
